# The Value of the “Trident Sign” and Flow Cytometry in Suspecting Spinal Cord Sarcoidosis: A Case Report and a Flow Chart of Diagnostic Imaging in Longitudinally Extensive Transverse Myelitis (LETM)

**DOI:** 10.7759/cureus.79227

**Published:** 2025-02-18

**Authors:** Maurizio Giorelli, Sergio Altomare, Ruggiero Leone, Rosario F Balzano, Silvio Orlando, Pasquale Di Fazio, Leonardo Santo

**Affiliations:** 1 Operative Unit of Neurology, "Dimiccoli" General Hospital, Barletta, ITA; 2 Operative Unit of Radiology, "Dimiccoli" General Hospital, Barletta, ITA; 3 Operative Unit of Thoracic Surgery, Mater Dei Hospital, Bari, ITA; 4 Operative Unit of Nuclear Medicine, "Dimiccoli" General Hospital, Barletta, ITA; 5 Operative Unit of Rheumatology, "Dimiccoli" General Hospital, Barletta, ITA

**Keywords:** cytofluorometry, longitudinally extensive transverse myelitis, magnetic resonance imaging, neurosarcoidosis, paraparesis

## Abstract

The diagnosis of sarcoidosis-related longitudinally extensive transverse myelitis (LETM) is challenging, requiring the exclusion of mimicking conditions along with histopathological confirmation of the diseases affecting the spinal cord. This report presents the case of a 68-year-old woman with complaints of low back pain and tingling dysesthesia in the limbs one month prior and finally developed paraparesis with the need for support from a third person for walking. Magnetic resonance imaging (MRI) of both the brain and spinal cord, total body computed tomography, lumbar puncture, flow cytometry of the cell sediment from cerebrospinal fluid (CSF), and whole-body 18-F-fluorodeoxyglucose positron emitting tomography (FDG-PET) assisted us to finalise the diagnosis. The presence of the “trident sign” in axial sequences of MRI is of exceptional value since it is a clue for sarcoidosis of the spinal cord. CSF flow cytometry may help clinicians to rule out lymphoma, and FDG-PET can help clinicians in identifying manifestations of sarcoidosis, which need to be biopsied so as to unveil the final diagnosis.

## Introduction

Sarcoidosis is an autoimmune disease characterised by the formation and development of non-caseating granulomas in affected organs. Although any tissue can be affected, the lungs are involved in approximately 95% of cases. The nervous system is usually involved as a secondary manifestation in 5-10% of cases and as a primary presentation in a minority of cases. Neurosarcoidosis (NS) has several imaging features and a wide range of clinical presentations, ranging from cranial neuropathy to paraparesis [1].

Sarcoidosis of the spinal cord is rare, occurring in approximately 1% of all cases, and even rarer as the initial manifestation. Due to the persistence and infiltrative nature of granulomas, spinal cord sarcoidosis may easily involve three or more vertebral segments (long extensive transverse myelitis, LETM) compromising the patient’s gait and autonomy. Accurate diagnosis is crucial to initiate effective immunosuppressant therapy, which is usually achieved through a wide, deep, and complex work-up. Along with specific pictures, magnetic resonance imaging (MRI) enhancement patterns such as the “trident sign” [2] and the “string of pearls” [3] signs have been found to be suggestive of spinal cord sarcoidosis. In the cerebrospinal fluid (CSF), lymphocyte pleocytosis, an increase in the number of proteins and oligoclonal bands are often reported but do not have specific pictures. The serum angiotensin-converting enzyme (ACE) is elevated in the blood serum but not in the CSF from up to 51% of NS patients [4].

In the absence of sensitive and specific non-invasive tests, the “not better explanation” criterion is conceived to be dangerous due to the lethality of mimicking diseases that might not be recognised [5]. Thus, the diagnostic path ends with biopsy procedures and histopathological studies to confirm the disease. Herein, we report a case of sarcoidosis-induced LETM associated with a literature review of diagnostic procedures and propose a diagnostic algorithm.

## Case presentation

A 68-year-old woman presented to our emergency department with the complaint of progressive paraparesis, which had been experiencing for a month, along with a sensation of numbness in all four limbs and intense burning pain in the lumbosacral area. Her personal and family medical history was unremarkable. She presented with spastic paraparesis, which allowed her to walk independently with support. Mild information ataxia was also observed. Her strength was rated 3+ for all lower limb movements. The right upper limb was also weak and rated as 3/5 for all movements. Deep tendon reflexes were brisk in the lower and upper right limbs and rated 3+. Search for the Babinski sign on the right disclosed extension. Vibration sensation was deficient from the hips down. Abdominal reflexes were absent. However, the pinprick sensation was unaffected. Subacute progressive lower-limb weakness with brisk reflexes may arise either from myelopathy or from a disease affecting the frontal lobe. 

**Figure 1:**
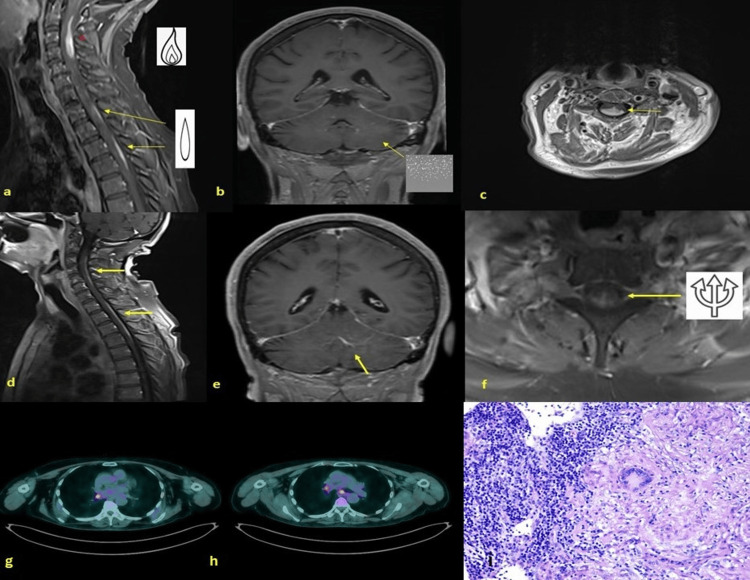
Imaging and histological studies Initial MRI showing “the flame” and the “elongated drops” signs (a; post-Gd-T1-w sagittal image) together with “sugar-like” lesions of leptomeningeal meninges (b; post-Gd-T1-w coronal image) and tumefactive appearance of the spinal cord (c; post-Gd-T1-w axial image) raised suspicion for solid tumour, satellite metastases and carcinomatous leptomeningitis. Later MRI (three months later) showed two LETMs (d; post-Gd-T1-w), contrast-enhancement of folia leptomeninges (e, post-Gd-T1-w), and the “trident” sign (f; post-Gd-T1-w). Post-Gd-T1-w: post-gadolinium-T1-weighed. 18-F-Fluorodexoxyglucose Positron Emitting Tomography (FDG-PET) demonstrating the presence of overactive lymph nodes at the mediastinal, hilar level of the right lower pulmonary bronchus and at subcarinal level (g,h). Biopsy of thoracic lymph nodes demonstrating non-necrotising granulomas characterised by epithelioid and giant cells surrounded by smaller mononuclear cells (i).

MRI of the spinal cord revealed two long lesions, which almost completely involved the posterior and lateral cord media from C3 to C6 and from D1 to D5 (Fig. 1a, 1c) [6]. On the brain MRI, some punctiform “sugar-like” areas of contrast enhancement affecting the superior cerebellar folia were seen. These findings suggested either a long-lasting infection or an immune-mediated disease of the central nervous system (CNS) [7,8]. Routine blood tests were normal, as were inflammatory biomarkers and both infection and autoimmune panels. Blood lymphocyte subpopulations showed a reduction in CD8 suppressors (10.45%, n.v.: 15-35) and an increase in the CD4/CD8 ratio (6.2, n.v.: 1.2-2.5). A diagnostic lumbar puncture demonstrated normal opening pressure, 80 cells/µl (85% lymphocytes, 15% plasma cells), 100 mg/dl proteins (n.v. up to 40 mg/dl), and normal glucose and lactate values. Oligoclonal bands were not observed. HIV, venereal disease research laboratory (VDRL), and *Treponema pallidum* haemagglutination (TPHA) tests were negative. IgM and IgG titres for *Borrelia burgdorferi *were normal. Polymerase chain reaction failed to detect the presence of DNA from all herpes viruses, including Coxsackie, enterovirus, JC virus, Parvovirus B19, Adenovirus, Parotite virus, *Neisseria meningitidis*, *Haemophilus influenzae*, *Listeria monocytogenes*,* E. coli* K1, and *Streptococcus pneumoniae*. Paraneoplastic and cell-surface-directed antibodies were also ruled out. Examination of the cell sediment from the CSF revealed the presence of lymphocytes, plasma cells, and histocytes, but not malignant cells. Anti-aquaporin-4 (AQP4)-IgG and anti-myelin oligodendrocyte glycoprotein (MOG)-IgG were evaluated using cell-based assay and were not detected in the serum. Total-body computed tomography (CT) ruled out the presence of solid malignant masses, abscesses, or adenopathies.

The "flame" appearance of the upper lesion within the spinal cord and the “elongated drops” shape of its underlying lesions could, in some way, mimic that of a solid tumour and satellite metastases [9,10]. However, homogeneous contrast enhancement, absence of intralesional inhomogeneity, absence of xanthochromia and malignant cells, and abundance of lymphocytes in the CSF sediment made this diagnosis unlikely. On the other hand, the reduction in CD8 suppressor, increase in the CD4/CD8 ratio, presence of lymphocytes, plasma cells, and histiocytes in the CSF, preserved glucose, and normal lactate levels indicated an inflammatory, possibly autoimmune disease [6,8]. Rheumatoid arthritis, Sjögren’s disease, systemic lupus erythematosus, and Bechet's disease were ruled out based on anamnesis, physical examination, and the absence of specific autoantibodies in the serum. Standing on the multifocal contrast enhancement of lesions involving either the brain or the spinal cord, a provisional diagnosis of acute disseminated encephalomyelitis (ADEM) was made, and a high-steroid schedule was initiated and later tapered to oral prednisone after discharge. At the clinical follow-up three months later, the patient had slightly improved but was still paraparetic and ataxic. Spinal cord MRI demonstrated further worsening of the known lesions, with further craniocaudal extension from C2 to C6 and from C7 to D5 (Fig. 1d). The lesions were intensely contrast-saturated and involved both posterior and lateral cords. In the coronal sequences, the spinal cord lesions had a typical appearance, resembling the one known as the "trident" sign and suggestive of pial and subpial involvement (Fig. 1f).

Finally, considering both the patient’s age and imaging findings, two possibilities still existed: NS and lymphoproliferative disease of the CNS [6]. Indeed, the "trident" sign has been detected in imaging of spinal cords from both these diseases [2,11] and the slight leptomeningeal involvement of the brain could fit well with each of them. ACE concentrations were normal (60.6 U/l; n.v.: 19.8-70.2). A flow cytometry was carried out on the cellular sediment of the CSF, which demonstrated a polyclonal population of lymphocytes (93% CD3+; 75% CD3+CD4+; 15% CD3+CD8+; CD3+ anti-TCR-α/β+; 92% CD5+; 2% CD19+; 2% CD20+; 4% CD16+CD56+). While substantially ruling out B-cell lymphoma, this mixed polyclonal population may be compatible with a granulomatous reaction segregated within the subarachnoid space [4,8]. Giemsa staining of cells collected from CSF sediment highlighted mononuclear cells with dispersed chromatin (MG and A De L, personal observations), a typical finding of activated immune cells. A total body contrast-enhanced CT scan was requested but was found to be irrelevant. We then performed total body 18-F-fluorodeoxyglucose positron emitting tomography (FDG-PET) which demonstrated the presence of overactive lymph nodes at the mediastinal and hilar levels of the right lower pulmonary bronchus and at the subcarinal level (Fig. 1g, 1h, 1i).

**Figure 2:**
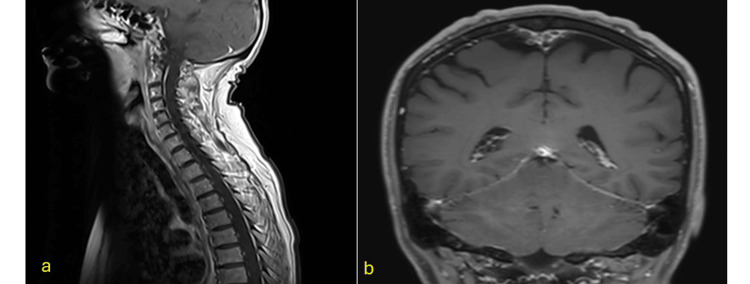
Imaging follow-up MRI follow-up at six months showed regression of intramedullary lesions (a). Contrast enhancement of cerebellar leptomeninges had also disappeared (b).

A subsequent lymph node biopsy was found to be consistent with sarcoidosis [12]. A final diagnosis of probable NS was made according to current diagnostic criteria [12,13,14], and a therapeutic schedule including azathioprine and infliximab was initiated [15]. Six months later, follow-up clinical and imaging examinations were performed. Paraparesis significantly improved and the patient could walk without assistance. MRI showed regression of the intramedullary lesions, which had reduced in length and volume (Fig. 2a). The contrast enhancement of the cerebellar leptomeninges had also disappeared (Fig. 2b).

## Discussion

Spinal sarcoidosis can manifest as intramedullary, intradural (though extramedullary), and epidural or vertebral body lesions. When leading to myelitis, sarcoidosis may present as a short discrete lesion confined to single or two vertebral segments or even involve the entire cord [16]. Inflammation may affect the posterior or anterior funiculi or be panmedullary [17]. MRI manifestations of the intramedullary lesions are categorised into four stages, starting (phase 1) with inflammatory involvement of leptomeninges, which couple with gadolinium enhancement of the dorsal pia mater, extending with infiltration of parenchyma (phases 2 and 3), and ending with atrophy of the spinal cord (phase 4) [18].

**Figure 3:**
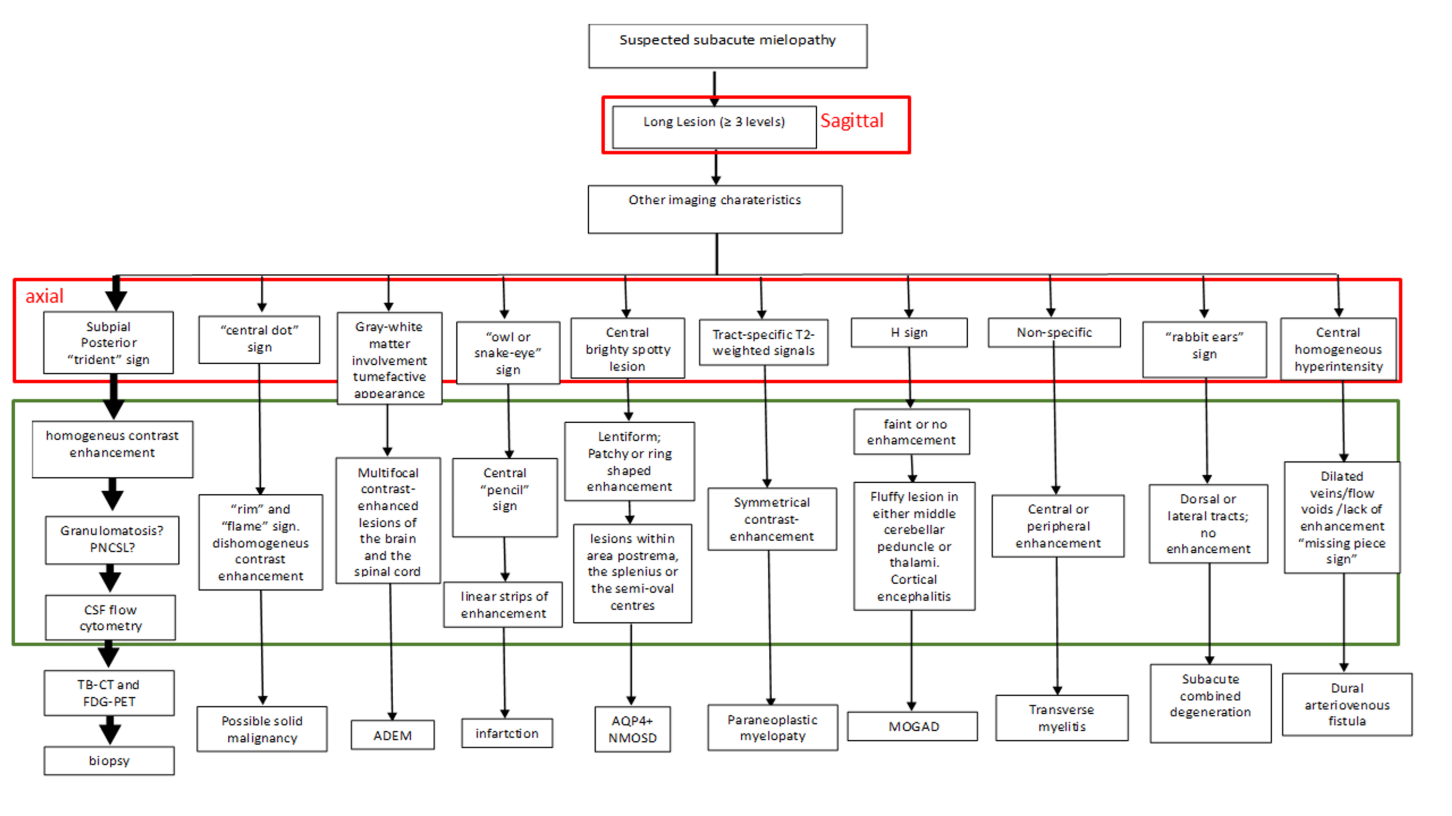
Flow chart of diagnostic imaging in LETM After detecting longitudinally extensive transverse myelitis (LETM) in the sagittal images, we activated to search for additional imaging features, which could guide our diagnostic process. We looked carefully at T2-w and FLAIR axial images together with brain sections and features of contrast enhancement. The “trident” sign revealed by post-Gd-T1-w axial images of the spinal cord, with contrast enhancement of leptomeninges from cerebellar folia, which prompted us to follow the diagnostic path of central nervous system (CNS) granulomatosis (heavy thick arrows). The red square represents disease-specific axial T2-w features. The green square represents other imaging features, which can be of fundamental help in the diagnostic process of each distinct disease.

LETM as the first manifestation of sarcoidosis in an otherwise healthy patient is rare [15]. Due to the severity of the clinical picture associated with sarcoidosis-related LETM, assessing the diagnosis and initiating therapy as soon as possible to avoid complete disability is mandatory. LETM has a broad differential diagnosis, including infectious agents (enteroviruses, cytomegaloviruses, dengue virus, human immunodeficiency virus, syphilis, and tuberculosis), systemic inflammatory diseases (sarcoidosis, Sjogren’s syndrome, connective tissue disease, systemic lupus erythematous disease, and scleroderma), or disorders within the CNS (multiple sclerosis, neuromyelitis optic spectrum disorder (NMOSD), paraneoplastic syndrome, vasculitis, and ADEM) [18,19]. When all other possible mimicking diseases are ruled out through a deep workup, clinicians are obliged to collect the involved tissue from the spinal cord to reach a “definite” diagnosis based on the current diagnostic criteria [14]. Alternatively, a biopsy of affected tissue outside the CNS can gain at least a diagnosis of “probable” sarcoidosis. This case of sarcoidosis-induced LETM prompted an investigation into refining a diagnostic algorithm. Sarcoidosis-induced LETM has been extensively described [15,19-29]. Among these, few described the “trident sign” in spinal cord diseases [2,7,11,30-32]. When myelopathy-related syndromes manifest, an MRI of the brain and spinal cord is required. A recent review described the imaging features of inflammatory myelopathies [7]. If a lesion extending for more than three metameres emerges on MRI, clinicians should focus on the differential diagnosis of LETMs. Axial sequences may be very helpful in such a diagnostic path, as they can unveil unique pictures and specific diseases. We drew a flowchart that might help identify specific patterns of myelopathies intersecting the identification of an LETM through sagittal MRI scans with axial sequences (Fig. 3).

Intramedullary sarcoidosis has a wide range of imaging manifestations, potentially involving the anterior or posterior funiculi. The most common picture is leptomeningeal enhancement with patchy involvement of the spinal cord [33]. This has been observed in most cases in the literature reviewed herein. On the other hand, a combination of linear dorsal subpial and central canal enhancement, namely, the “trident” sign, is very suggestive of leptomeningeal sarcoidosis but an uncommon finding [30,31,32]. Besides intramedullary sarcoidosis, the “trident sign” has been observed in spinal cord imaging of B-cell lymphoma [11]. In our case, the finding of a polyclonal lymphocytic population in the CSF and an increased CD4/CD8 T cell ratio in the blood ruled out the diagnosis of lymphoma [34] and strengthened the suspicion of granulomatosis. A whole-body FDG-PET scan, undertaken to detect hypermetabolic thoracic lymph nodes, directed the choice of biopsy site. It is noteworthy that 95% of patients with sarcoidosis-associated myelitis present with abnormal findings on 18FDG-PET scans [16]. This is significant because the final diagnosis of NS requires a biopsy and histological confirmation [13,14], a procedure that carries a risk of serious complications when the disease is predominantly confined to the spinal cord. Although limited to the level of “probable” sarcoidosis, the detection of typical noncaseating granulomas outside the CNS in the context of a clinical and imaging picture consistent with spinal cord sarcoidosis, could justify initiating specific therapy. The development of new diagnostic criteria incorporating clinical findings, imaging results, and CSF characteristics may be helpful [12,14].

Prompt initiation of pharmacological treatment is essential once sarcoidosis has been diagnosed, especially in cases of extrapulmonary involvement [35]. The cornerstone for the treatment of all types of sarcoidosis is prednisone at a usual dosage of 20-40 mg/day. Second-line therapies, including methotrexate, azathioprine, leflunomide, and mycophenolate, are critical as corticosteroid-sparing. TNF inhibitors, such as infliximab, showed significant effectiveness in addition to fewer side effects. In our patient, infliximab substantially reduced intramedullary inflammation and facilitated recovery from neurological deficits within six months. According to current treatment protocols, a combined regimen of glucocorticoids and steroid-sparing immunosuppressants, maintained for at least one year, may help prevent relapses in NS [1].

## Conclusions

Diagnosing NS can be particularly challenging when the spinal cord is involved, and obtaining a biopsy carries a high risk of severe complications. The presence of the “trident sign” in axial sequences of MRI is of exceptional value since it is a clue for sarcoidosis of the spinal cord. Clinicians should be aware that MRI images reveal LETM with involvement of the posterior funiculi and central channel, possibly along the lateral sides of the spinal cord. This is because the picture may be easily related to spinal cord sarcoidosis. CSF flow cytometry should be performed whenever a long inflammatory lesion is detected within the spinal cord, and the differential diagnosis should be matched against CNS lymphoma. In addition, FDG-PET can assist clinicians in identifying extra-CNS manifestations of sarcoidosis, especially when contrast-enhanced total-body CT results are irrelevant. Therefore, this approach facilitates the identification of organs for biopsy, improving diagnostic accuracy.

## References

[1] Kidd DP (2020). Neurosarcoidosis: clinical manifestations, investigation and treatment. Pract Neurol.

[2] Zalewski NL, Krecke KN, Weinshenker BG, Aksamit AJ, Conway BL, McKeon A, Flanagan EP (2016). Central canal enhancement and the trident sign in spinal cord sarcoidosis. Neurology.

[3] Li X, Gomez LM, Al Masry M, Basuroski ID (2022). "String of pearls" in spinal cord sarcoidosis. Ann Neurol.

[4] Arun T, Pattison L, Palace J (2020). Distinguishing neurosarcoidosis from multiple sclerosis based on CSF analysis: A retrospective study. Neurology.

[5] Shen J, Lackey E, Shah S (2023). Neurosarcoidosis: diagnostic challenges and mimics a review. Curr Allergy Asthma Rep.

[6] Lopez Chiriboga S, Flanagan EP (2021). Myelitis and other autoimmune myelopathies. Continuum (Minneap Minn).

[7] Cacciaguerra L, Sechi E, Rocca MA, Filippi M, Pittock SJ, Flanagan EP (2022). Neuroimaging features in inflammatory myelopathies: a review. Front Neurol.

[8] Reske D, Petereit HF, Heiss WD (2005). Difficulties in the differentiation of chronic inflammatory diseases of the central nervous system--value of cerebrospinal fluid analysis and immunological abnormalities in the diagnosis. Acta Neurol Scand.

[9] Grillo A, Capasso R, Petrillo A, De Vita F, Conforti R (2019). An intramedullary "flame" recognized as being an intramedullary spinal cord metastasis from esophageal cancer. J Radiol Case Rep.

[10] Fanous AA, Olszewski NP, Lipinski LJ, Qiu J, Fabiano AJ (2016). Idiopathic transverse myelitis mimicking an intramedullary spinal cord tumor. Case Rep Pathol.

[11] Griffin KJ, Toledano M, Flanagan EP (2023). “Trident sign” in primary CNS B-cell spinal cord lymphoma. Neurology.

[12] Bradshaw MJ, Pawate S, Koth LL, Cho TA, Gelfand JM (2021). Neurosarcoidosis: pathophysiology, diagnosis, and treatment. Neurol Neuroimmunol Neuroinflamm.

[13] Lin J, Pulst-Korenberg J, Zamvil SS, Graves J, Newsome SD, Amezcua L (2024). Tuberculous meningitis or neurosarcoidosis-a diagnostic quandary. From the National Multiple Sclerosis Society Case Conference Proceedings. Neurol Neuroimmunol Neuroinflamm.

[14] Stern BJ, Royal W 3rd, Gelfand JM (2018). Definition and consensus diagnostic criteria for neurosarcoidosis: from the Neurosarcoidosis Consortium Consensus Group. JAMA Neurol.

[15] Kumar A, Bai R, Sanjna F (2024). Longitudinally extensive transverse myelitis as an initial manifestation of sarcoidosis: a rare case and its management. Clin Case Rep.

[16] Nolte JY, Ten Dam L, van de Beek D, Brouwer MC (2022). Clinical characteristics and outcome of neurosarcoidosis-associated myelitis: a retrospective cohort study and review of the literature. Eur J Neurol.

[17] Soni N, Bathla G, Pillenahalli Maheshwarappa R (2019). Imaging findings in spinal sarcoidosis: a report of 18 cases and review of the current literature. Neuroradiol J.

[18] Junger SS, Stern BJ, Levine SR, Sipos E, Marti-Masso JF (1993). Intramedullary spinal sarcoidosis: clinical and magnetic resonance imaging characteristics. Neurology.

[19] Cicia A, Nociti V, Bianco A, De Fino C, Carlomagno V, Mirabella M, Lucchini M (2022). Neurosarcoidosis presenting as longitudinally extensive myelitis: diagnostic assessment, differential diagnosis, and therapeutic approach. Transl Neurosci.

[20] Chaubey M, Meena K, Singh T (2024). Neurosarcoidosis: an under-diagnosed cause of myelopathy. J Family Med Prim Care.

[21] Touri J, Bali A, Poma JF (2024). Sarcoidosis presenting as longitudinally extensive transverse myelitis: a case report. J Belg Soc Radiol.

[22] Patel M, Shiwlani S, Kachhadia MP, Abdalla M, Samreen I, Mohamed AS, Nasir H (2024). Neurosarcoidosis and transverse myelitis: life-threatening manifestations of sarcoidosis. Cureus.

[23] Abrantes FF, Moraes MP, Pedroso JL, Barsottini OG (2023). Diffuse leptomeningeal enhancement in neurosarcoidosis-related longitudinally extensive myelitis. Arq Neuropsiquiatr.

[24] Rodrigues RA, Alves T, Sousa JA, Jorge A, Geraldo A (2023). Longitudinally extensive transverse myelitis as a first manifestation of sarcoidosis. Cureus.

[25] Cassimatis N, Hong E, Trippiedi A, Lauer SA (2023). Neurosarcoidosis presenting as longitudinally extensive transverse myelitis and orbital mass: a case report. Cureus.

[26] Al Malik YM (2020). Isolated neurosarcoidosis mimicking multiple sclerosis. Neurosciences (Riyadh).

[27] Scott AM, Yinh J, McAlindon T, Kalish R (2018). Two cases of sarcoidosis presenting as longitudinally extensive transverse myelitis. Clin Rheumatol.

[28] Gupta SS, Shankar S, Stein E, Khasani S (2017). Neurosarcoidosis-induced longitudinal extensive transverse myelitis. Neurol India.

[29] Gibbons E, Whittam D, Jacob A, Huda S (2021). Images of the month 1: Trident sign and neurosarcoidosis. Clin Med (Lond).

[30] Bala M, Saucedo M, Bandeo L (2020). Trident sign in spinal cord neurosarcoidosis [Article in Spanish]. Rev Neurol.

[31] Jolliffe EA, Keegan BM, Flanagan EP (2018). Trident sign trumps Aquaporin-4-IgG ELISA in diagnostic value in a case of longitudinally extensive transverse myelitis. Mult Scler Relat Disord.

[32] Flanagan EP, Kaufmann TJ, Krecke KN (2016). Discriminating long myelitis of neuromyelitis optica from sarcoidosis. Ann Neurol.

[33] Bromberg JE, Breems DA, Kraan J (2007). CSF flow cytometry greatly improves diagnostic accuracy in CNS hematologic malignancies. Neurology.

[34] Gerke AK (2020). Treatment of sarcoidosis: a multidisciplinary approach. Front Immunol.

[35] Gelfand JM, Bradshaw MJ, Stern BJ (2017). Infliximab for the treatment of CNS sarcoidosis: a multi-institutional series. Neurology.

